# Proteomic dataset for altered glycoprotein expression upon *GALNT3* knockdown in ovarian cancer cells

**DOI:** 10.1016/j.dib.2016.05.060

**Published:** 2016-05-30

**Authors:** Razan Sheta, Florence Roux-Dalvai, Christina M. Woo, Frédéric Fournier, Sylvie Bourassa, Carolyn R. Bertozzi, Arnaud Droit, Dimcho Bachvarov

**Affiliations:** a Department of Molecular Medicine, Laval University, Québec PQ, Canada; b Centre de recherche du CHU de Québec, L’Hôtel-Dieu de Québec, Québec PQ, Canada; c Centre de recherche du CHU de Québec, CHUL, Québec PQ, Canada; d Department of Chemistry, Stanford University, Stanford, CA, USA; e Howard Hughes Medical Institute, Stanford University, Stanford, CA, USA

**Keywords:** GALNT3, Glycosylation, Ac4GalNAz labeling, Label free quantification, NetOGlyc and NetNGlyc prediction analysis, Glycoproteomics

## Abstract

This article contains raw and processed data related to research published in “*Role of the polypeptide N-acetylgalactosaminyltransferase 3 in ovarian cancer progression: possible implications in abnormal mucin O-glycosylation*” [1]. The data presented here was obtained with the application of a bioorthogonal chemical reporter strategy analyzing differential glycoprotein expression following the knock-down (KD) of the *GALNT3* gene in the epithelial ovarian cancer (EOC) cell line A2780s. LC-MS/MS mass spectrometry analysis was then performed and the processed data related to the identified glycoproteins show that several hundred proteins are differentially expressed between control and *GALNT3* KD A2780s cells. The obtained data also uncover numerous novel glycoproteins; some of which could represent new potential EOC biomarkers and/or therapeutic targets.

**Specifications Table**

**Table:** 

Subject area	Biology
More specific subject area	Oncology, Proteomics
Type of data	Table, figure
How data was acquired	Mass spectrometry, Orbitrap Fusion mass spectrometer (Thermo Fisher Scientific, San Jose, CA, USA)
Data format	Raw, analyzed
Experimental factors	Metabolite labeling of glycoproteins from extracellular/membrane bound, cytoplasmic and nuclear fractions of ovarian cancer cells, followed by trypsin digestion and glycoproteomics enrichment using Click Chemistry, and subjected to nanoLC and analyzed by ESI MS/MS.
Experimental features	Subcellular fractionation, ESI MS/MS peptide identification, data analysis in MaxQuant followed by data predictions using the NetOGlyc 4.0 and NetNGlyc 1.0 servers, and enrichment analysis using GO Consortium for enrichment analysis.
Data source location	Quebec City, Canada
Data accessibility	Data is with this article

**Value of the data**

• The presented list of differentially regulated glycoproteins identified upon *GALNT3* KD in EOC cells could represent novel putative biomarkers/molecular targets involved in EOC metastasis and thus the data presented here can be a useful resource to examine some of these biomarkers.
• The metabolic labeling approach applied in this study followed by the MS analysis could be a useful tool/guide for the quantification and the identification of glycoproteins from different cell lines.
• The data presented herein provide a comprehensive list of newly identified glycoproteins, which strongly suggests that the metabolic labeling approach applied can essentially increase the magnitude of recognized glycoproteins by comparing to organism-specific database for a more complete level of identification.

## 1 Data

The datasets provided in this article represent the entire list of identified glycoproteins after GalNAz metabolic labeling in both control and *GALNT3* KD A2780s cells, in addition to the processed data identifying the quantitatively significant list of differentially regulated proteins between the control and *GALNT3* KD cells. Data represented here also include comparative analysis of identified glycoproteins with previously published glycoproteins’ data. This was also supported by predictive analysis performed by investigating for possible glycosylation sites from the list of the identified proteins for further confirmation. Finally, data of protein enrichment analysis performed were included as a representation of the cellular localization of the assigned glycoproteins from our list of differentially regulated proteins.

## 2 Experimental design, materials and methods

**Fig. 1:**
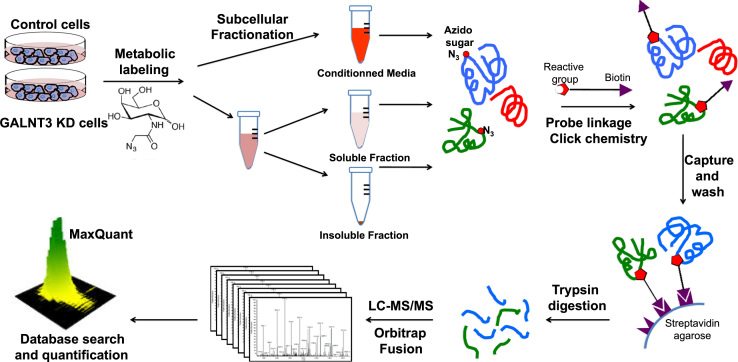
Schematic overview of the glycoproteomic workflow used.

**Fig. 2:**
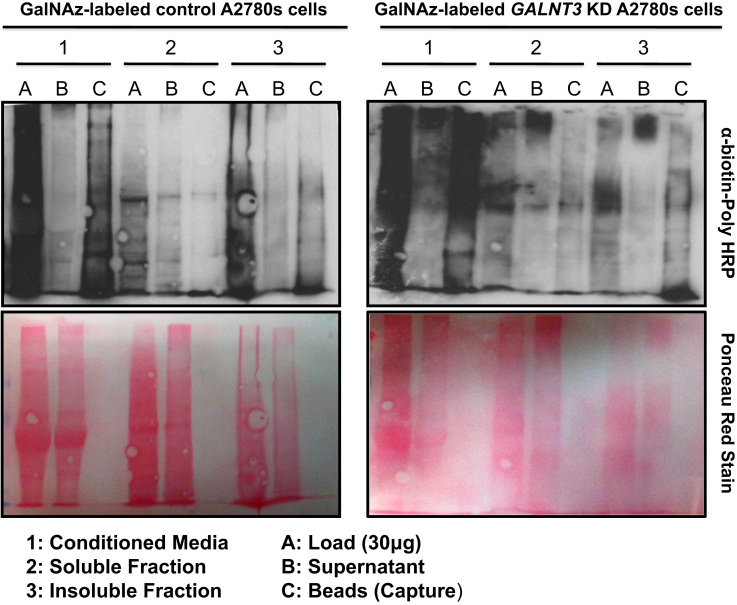
Western blot analysis of glycoproteins enrichment in control and *GALNT3* KD A2780s cells. Whole-cell lysates labeled with 100 μM Ac_4_GalNAz were incubated with a biotinylated bioorthogonal probe. Anti-biotin signal was checked before affinity-capture (Load) and after affinity-capture on the fraction not bound to the beads (Supernatant) and on the fraction that included the bead after washing (Capture).

We applied a bioorthogonal chemical reporter strategy [2], [3] for analyzing differential glycoprotein expression following *GALNT3* KD in the EOC cell line A2780s. Fig. 1 represents a schematic overview of the glycoproteomic workflow used. The method is explicitly used for metabolically labeling glycans with a monosaccharide precursor attached to a functional azido group [4]. Control and *GALNT3* KD A2780s cells were separately labeled with tetraacetylated N-azidoacetylgalactosamine (Ac_4_GalNAz) or tetraacetylated N-acetylgalactosamine (Ac_4_GalNAc, negative control). The labeled control and *GALNT3* KD A2780s cells were then subjected to subcellular fractionation (conditioned media fraction, soluble fraction and insoluble fraction) followed by glycoprotein enrichment (Fig. 1). A Western blot analysis was performed to examine the enrichment efficiency (Fig. 2). Trypsin digestion was then performed and the released peptides were analyzed by LC–MS/MS. For each sample labeled with Ac_4_GalNaz, three technical replicates were performed in order to get statistical values on intensity measurements, while single injections of Ac_4_GalNAc samples were done for evaluation of non-specific binding on streptavidin-agarose resin.

Supplementary Table 1 displays the total number of proteins identified in the three subcellular fractions of the control and *GALNT3* KD A2780s cells cultured with Ac_4_GalNAz, as well as the subtracted proteins, exclusively found in the Ac_4_GalNAc (negative control) fraction. Analyses of these data using the NetOGlyc 4.0 and the NetNGlyc 1.0 servers generated lists of proteins with predicted O- and N-glycosylation sites (see Supplementary Table 2). Additionally, Supplementary Table 3 contains a list of proteins identified in our study that have been previously characterized as glycoproteins in the literature.

**Fig. 3:**
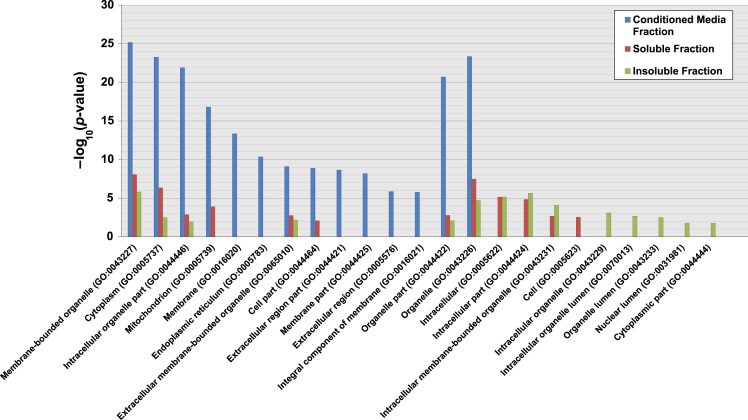
GO cellular component analysis of significantly enriched proteins found upon *GALNT3* KD. Bar graphs showing the cellular component GO terms that are significantly enriched from the differentially regulated proteins in our study, compared to the entire human proteome. Data were submitted to the GO Consortium for enrichment analysis [20]. The analysis was performed on the differentially regulated proteins identified from each of the three fractions: Conditioned media fraction (blue bars), Soluble fraction (red bars) and Insoluble fraction (green bars). All identified proteins annotated with GO cellular component terms were compared against the annotated human proteome. The enrichment *p*-value (≤0.05) of each term was transformed to a –log_10_ (*p*-value).

The MaxQuant software and Andromeda search engine (included in MaxQuant) [5], [6] were consecutively used to generate a list of differentially regulated proteins identified in the three A2780s subcellular fractions upon *GALNT3* KD, as based on the following criteria: Welch test *p*-value ≤0.05 and fold change in relative expression of ≥2 similar to that applied in [7,8] (see Supplementary Table 4). Cellular component Gene Ontology (GO) analysis of the differentially regulated glycoproteins identified between the control and *GALNT3* KD A2780s cells was additionally performed on each of the identified fractions (conditioned media fraction, cytosolic fraction and nuclear fraction), and data were compared to the entire human proteome using the GO Consortium for enrichment analysis (Fig. 3).

## 3 Chemical glycoproteomics enrichment using click chemistry

The first part of the platform protocol applied in this study represents a method used to metabolically label glycoproteins in cell culture, as described in [1]. Briefly, cells were separately labeled with Ac_4_GalNAz and Ac_4_GalNAc (Fig. 1). We started by isolating and enriching glycoproteins from different biological fractions, including proteins secreted into the media (conditioned media fraction), as well as proteins enriched in the cytosolic and nuclear and fractions (soluble and insoluble fractions respectively), as shown in [1].

The next step of the protocol was the chemical glycoproteomics enrichment procedure, which included tagging glycoproteins with Click Chemistry (Fig. 1). The Copper-Catalyzed Azide-Alkyne Cycloaddition (CuAAC) enrichment was performed, as previously described [9], [10]. GalNAz and GalNAc labeled cell fractions were divided into aliquots, each containing 3 mg of protein. Click-chemistry reagents (200 µM alkynyl biotin probe, 300 µM copper sulfate, 600 µM BTTP, and 2.5 mM sodium ascorbate) were pre-mixed and added, and the reaction was incubated for 3.5 h at 24 °C. Proteins were precipitated, resuspended and then solubilized as previously described [9], [10]. Briefly, protein pellets were resuspended in 400 μl 1% RapiGest/PBS and solubilized by probe sonication. Streptavidin-agarose resin was first washed with PBS and then added to the samples, and the resulting mixture was incubated for 12 h at 24 °C with rotation. The beads were then pelleted by centrifugation, and the supernatant containing uncaptured proteins was removed as a separate fraction (Fig. 1). The beads were then washed with 1% Rapigest, 6 M urea and PBS; beads were then pelleted by centrifugation and resuspended in PBS. Samples were subjected to reduction and alkylation, as previously described [9], [10]. Briefly, proteins were reduced by the addition of 5 mM DTT followed by alkylation completed by the addition of 10 mM iodoacetamide. Trypsin was then added to the slurry of beads and the resulting mixture was incubated for 12 h at 37 °C. The beads were pelleted and the supernatant digest was collected (Fig. 1). The trypsin fraction was concentrated to dryness using a Speedvac set to 40 °C. Samples were desalted by ZipTip P10 for subsequent MS analysis (Fig. 1).

## 4 Western blot analysis

Western blot analyses were performed on protein lysates collected from both the control and *GALNT3* KD A2780s EOC cells. Whole-cell lysates labeled with 100 μM Ac_4_GalNAz were incubated with a biotinylated bioorthogonal probe. Biotinylated glycoproteins were enriched from the supernatant by affinity-capture with streptavidin–agarose beads. To each aliquot collected during the enrichment procedure, 3 μl of 4X SDS buffer was added and the aliquots were loaded to 5% polyacrylamide gels. Proteins were then transferred to nitrocellulose membranes, which were consecutively incubated with Ponceau stain (Fig. 2). The membranes were then blocked with 2% bovine serum albumin in Tris-buffered saline with 0.1% Tween-20 for 1 h at 24 °C with gentle shaking and washed 3× with PBS-Tween. The blots were stained with streptavidin–HRP (1:1000) (Pierce, Streptavidin Poly-HRP) overnight at 4 °C with gentle shaking. Upon washing with PBS-Tween, the membranes were developed using the ECL Chemiluminescent Substrate (OriGene). Fig. 2 shows a Western blot demonstrating the incorporation of GalNAz into glycoproteins from protein lysates collected from the three fractions (conditioned media fraction, soluble and insoluble fractions). Anti-biotin signal was checked before affinity-capture (Load) and after affinity-capture on the fraction not bound to the beads (Supernatant) and on the fraction that included the bead after washing (Capture), as performed in [9] (Fig. 2).

## 5 Database searching and label free quantification

The released glycopeptides were consecutively analyzed by reversed-phase nanoflow liquid chromatography coupled to a Thermo LTQ–Orbitrap fusion mass spectrometer, as described in [1] (also see Fig. 1). Spectra were searched against a human proteins database (Uniprot Complete Proteome – taxonomy Homo sapiens – 69165 sequences) using the Andromeda module of MaxQuant software v. 1.5.2.8 [6], [11]. Trypsin/P enzyme parameter was selected with two possible missed cleavages. Carbamidomethylation of cysteins was set as fixed modification, and methionine oxidation and acetylation of protein N-terminus were set as variable modifications, similar to that applied in [12]. Search mass tolerances were defined at 5 ppm and 0.6 Da for MS and MS/MS respectively. For protein validation, a maximum false discovery rate of 1% at peptide and protein level was used based on a target/decoy search. MaxQuant was also applied for Label Free Quantification (LFQ), as shown in [13]. The ‘match between runs’ option was used with a 20 min value as the alignment time window and 3 min as match time window. Only unique and razor peptides were used for quantification. The LFQ intensity values (normalized values) extracted by MaxQuant for each protein in each sample replicate were used to calculate a ratio between two samples to compare as well as a *p*-value based on a Welch׳s test similar to that applied in [14] (see Supplementary Table 1). When LFQ intensity values were missing, they were replaced either by the average of the values of the two other replicates, or, if less than two replicate values were present, by a noise value corresponding to the first percentile of LFQ values of all proteins of the sample replicate, as described in [14] (see Supplementary Table 1). A protein was considered as quantifiable only if at least two of the replicate values in one of the two samples to compare were present before performing the missing values replacement (Supplementary Table 1).

Differentially regulated proteins between *GALNT3* KD and control A2780s cells were defined based on the following selection criteria: 2-fold change in expression level and *t*-test *p*-value cutoff of ≤0.05, as described [15], [16], [17], [18]. A *z*-score was also calculated for each protein based on the statistical approach described in [16], where *z*-score={(Welch *t*-test difference)−Median (Welch *t*-test difference) for all quantified proteins}/Standard deviation (Welch *t*-test difference) for all quantified proteins as described in [16].

To classify proteins as variant, different combinations of stringent filtering criteria were tested (Supplementary Table 4):

1. Filtering 1 (Welch *p*-value ≤0.05 and fold change of ≥2)
2. Filtering 2 (Welch *p*-value ≤0.05 and *z*-score >1)

The list of the differentially regulated proteins is presented in Supplementary Table 4.

## 6 Bioinformatic annotation & analysis

### 6.1 Glycoprotein prediction analysis

The NetOGlyc 4.0 server (http://www.cbs.dtu.dk/services/NetOGlyc-4.0/) was used to identify the O-glycosylated proteins identified from our control and *GALNT3* KD A2780s EOC cells, (using G-score >0.5), as described in [5]. The identified predicted O-glycosylated proteins are listed in Supplementary Table 2.

The NetNGlyc 1.0 server (http://www.cbs.dtu.dk/services/NetNGlyc/) was used to find the N-glycosylated proteins identified from our control and *GALNT3* KD A2780s EOC cells. Sequences having N-glycosylation potential >0.5 were considered as cut-off value [19]. The identified predicted N-glycosylated proteins are listed in Supplementary Table 2.

An additional prediction approach used in our study was essentially focused on reviewing recent literature for previously identified glycoproteins. The list of proteins identified and compared to the literature data is found in Supplementary Table 3.

### 6.2 Protein enrichment analysis

GO enrichment analysis of the cellular localization of the identified differentially regulated proteins was performed using information from AmiGO (http://amigo.geneontology.org). The GO term enrichment tool was used to determine the observed level of annotations for the set of proteins from our study and determine the significance in the context of all proteins annotated in the human proteome [20]. Data was presented as percent of enrichment. The GO terms found to be over/under represented by a two-tailed Fisher Exact test with a *p-*value ≤0.05 were presented, *p*-values were corrected using Bonferroni statistics correction (See Fig. 3 in [1] and Supplementary Table 5). *P-*values were additionally transformed to scores (–log_10_ (*p*-value)), to determine whether the fold enrichment is significant based on the relative abundance of each GO term in our data sets (*p*≤0.05 is considered significant) (see Fig. 3 and Supplementary Table 5). The GO terms based on the gene list of our study were compared to the background distribution of annotation based on the genes in the whole genome that are annotated to the GO Term similar to that applied in [20] (see Fig. 3 and Supplementary Table 5).

## Footnotes

Appendix A Supplementary data associated with this article can be found in the online version at doi:10.1016/j.dib.2016.05.060.
