# Supplementary material for: Proteomic dataset for altered glycoprotein expression upon GALNT3 knockdown in ovarian cancer cells
Source: Data Brief. 2016 May 30;8:342–9. doi: 10.1016/j.dib.2016.05.060 (PMC4908283; doi:10.1016/j.dib.2016.05.060)
Supplement: Supplementary file 1 — Supplementary material [file mmc1.docx]

All authors declare no conflict of interests.
